# Effect of the P/Al Molar Ratio and Heating Rate on the Composi-Tion of Alumino-Phosphate Binders

**DOI:** 10.3390/ma15062337

**Published:** 2022-03-21

**Authors:** Grégory Tricot, Hanyu Hu, Amélie Beaussart, Ismérie Fernandes, Clément Perrot

**Affiliations:** University Lille, CNRS, UMR 8516-LASIRE-Laboratoire de Spectroscopie pour les Interactions, La Réactivité et L’Environnement, F-59000 Lille, France; hanyu.hu.etu@univ-lille.fr (H.H.); amelie.beaussart.etu@univ-lille.fr (A.B.); ismerie.fernandes.etu@univ-lille.fr (I.F.); clement.perrot.etu@univ-lille.fr (C.P.)

**Keywords:** phosphate binder, phosphate solutions, solid state NMR

## Abstract

New insights into the chemistry of alumino-phosphate solutions are provided in this contribution. In a first part, a solution with a P/Al molar ratio of 3.2 was prepared for the first time. The binders obtained at 500 and 700 °C were compared to those obtained with the 3 and 3.5 P/Al molar ratio solutions in order to determine the impact of moderate P_2_O_5_ excess on the final phosphate ceramic nature. In a second part, the widely used P/Al = 3 solution was heat-treated at 500 °C using different heating rates (0.2, 1, and 10 °C/min) to determine how this parameter modifies the final phosphate ceramic composition. Our data show that moderate P_2_O_5_ excess is sufficient to obtain binders with a high amount of stable cubic aluminium metaphosphate compound at 700 °C but not at 500 °C, where significant P_2_O_5_ excess is mandatory. We also show that slow heating favors the formation of cubic aluminium metaphosphate compound at 500 °C.

## 1. Introduction

Alumino-phosphate binders have been widely used in the development of refractories materials [1,2,3,4] acid–base cements [5] and protective coatings [6,7,8], due to their high bonding strength, abrasion resistance, and very high temperature stability. Those binders are generally prepared by heat-treating alumino-phosphate solutions that are obtained by dissolving Al_2_O_3_ or Al(OH)_3_ in diluted phosphoric acid solutions. Such solutions, characterised by their P/Al molar ratio, their phosphorus concentration [P], and the presence of additives, have also been widely investigated [1,2,3,4,5,6,7,8,9,10,11,12,13]. By heating, the alumino-phosphate species of the solutions react to form hydrated alumino-phosphate compounds (Al(H_2_PO_4_)_3_, × H_2_O and AlH_2_P_3_O_10_, × H_2_O) around 150–350 °C. Then, these hydrated compounds progressively condensate to form anhydrous aluminium metaphosphate Al(PO_3_)_3_ in a monoclinic structure (space group P2_1_/a), denoted as Al(PO_3_)_3_ [B]. At very high temperatures, this compound is replaced by the cubic aluminium metaphosphate structure (space group I43d), denoted as Al(PO_3_)_3_ [A]) [9] (Table 1).

The presence of Al(PO_3_)_3_ [A] is beneficial for the development of binders because this compound is stable and only melts at temperatures higher than 1200 °C. Several studies have been performed on solutions with a P/Al molar ratio of 3 because they present a similar P/Al ratio than the final Al(PO_3_)_3_ [A] phase [1,2,3,4,7,8,9,10,11,12,13,14]. Solutions with a P/Al ratio lower than 3 produce binders composed of Al(PO_3_)_3_ but also AlPO_4_ compounds [3,4]. However, these solutions are known to be quickly affected by precipitation (a white precipitate is observed at the bottom of the bottle after a few months of storage), thus hindering their uses in industrial applications. Solutions with a P/Al ratio higher than 3 contain phosphoric acid in excess (not participating in the formation of the Al(PO_3_)_3_ compounds) and have not been widely studied. However, we showed, in a previous investigation, that Al(PO_3_)_3_ [A] appears at lower temperature with a P/Al = 3.5 solution, which can be considered as an advantage compared to the classic formulation [10]. We also showed that the binders contain residual phosphoric acids (H_3_PO_4_, H_4_P_2_O_7_, or HPO_3_, denoted in the following as HPO_n_). This can be considered as a drawback compared to the classic formulation. However, during heating, orthophosphoric acid (H_3_PO_4_) will progressively condensate to H_4_P_2_O_7_, HPO_3_, and P_2_O_5_ to finally evolve from the materials at higher temperatures. In all the previously mentioned studies, simple or complex thermal treatments were used [1,2,3,4,5,6,7,8,9,10,11,12,13,14] but many papers only referred to the final curing temperature [1,4,11,12,14] with no real consideration for the effect of the heating rate used to reach this temperature. When the heating rate is mentioned [2,3,7,8,9,13], this parameter does not seem to be optimised, although it can be considered a key parameter.

The X-ray diffraction (XRD) technique has been the technique of choice for the investigation of these systems [1,2,3,9,12,13]. However, the efficiency of solid-state magic angle spinning nuclear magnetic resonance (MAS-NMR) spectroscopy has been clearly demonstrated in recent papers [10,11,14]. Indeed, ^31^P and ^27^Al NMR were able to confirm and complete the data obtained by XRD. NMR is able to highlight the presence of insensitive-XRD residual phosphoric acids and/or amorphous compounds through very narrow and/or broad peaks in the ^31^P NMR spectra. In addition, a qualitative analysis of the materials can be quickly performed using the chemical shift values observed for the main alumino-phosphate phases in previous studies (Table 2) [10,15,16]. Quantification can also be quickly and efficiently achieved through the decomposition of the quantitative ^31^P NMR spectra [10,11].

This work aims to complete the investigation of the alumino-phosphate solution thermal evolution. In a first part, a new solution with a P/Al molar ratio of 3.2 will be prepared and investigated for the first time. The binders obtained at 500 and 700 °C with this solution will be compared to the binders prepared with the widely used P/Al = 3 and the previously investigated (P/Al = 3.5 [10]) solutions. Therefore, the advantages/drawbacks of moderate P excess will be compared to the advantages/drawbacks previously observed in case of the P/Al = 3.5 solution. In a second part, different heating rates (0.2, 1, and 10 °C/min) will be applied to the widely used P/Al = 3 solution to prepare binders at 500 °C. The comparison between the binders nature will allow concluding about the importance of the heating rate parameter in the thermal treatments. In addition to standard XRD experiments, 1D (and correlation) MAS-NMR will be used in this study to provide quick and efficient qualitative and quantitative analysis of the prepared binders.

## 2. Experimental

Three alumino-phosphate solutions with P/Al molar ratio of 3, 3.2, and 3.5 were prepared by introducing appropriate amounts of Al(OH)_3_ in a mixture of 50 mL of 85% H_3_PO_4_ solutions and 50 mL of distilled water (Table 3). The mixtures were then stirred and refluxed for 20–45 min before becoming completely clear [2,10]. The density of each solution was determined using a 50 mL volumetric flask and is given with an error of +/−0.005. The solutions were stored at room temperature in glass bottles.

To investigate the thermal evolution of these solutions, 2–3 g of solution were placed in a Pt-Au crucible and heat-treated under air in an electric muffle furnace (NABERTHERM L3/12) following different protocols. In the first part of this study, the three solutions (P/Al = 3, 3.2, and 3.5) were heat-treated for 1 h at 500 or 700 °C using a heating rate of 1 °C/min in order to determine the effect of the P/Al molar ratio on the binder’s nature. In the second part of this study, the effect of the heating rate on the binder’s nature was investigated with two sets of experiments performed at 500 °C on the solution with a P/Al molar ratio of 3. In the first set, all the experiments ended with a 1 h dwell time at 500 °C. The total treatment times were thus 41, 9, and 1 h and 48 min for the 0.2, 1, and 10 °C/min heating rates, respectively. In the second set, all the experiments presented a similar total duration (41 h) thanks to different dwell times (1 h, 33 h, and 40 h and 12 min for the 0.2, 1, and 10 °C/min heating rates, respectively). These two sets of experiments allowed to distinguish between the kinetic effect (due to the different heating rates) and the total thermal treatment time.

The resulting powders were analysed by XRD and solid state MAS-NMR. The XRD experiments were performed on a Bruker D8 Advance diffractometer equipped with an energy dispersion detector solX. The acquisitions were recorded between 10 and 80°, with 0.02° scan step and 1 s of step time. The ^31^P and ^27^Al MAS-NMR experiments were performed on a 9.4 T Bruker spectrometer equipped with a 4 mm probe operating at a spinning frequency of 12.5 kHz. The ^27^Al MAS-NMR were acquired at 104 MHz with a radio-frequency field (rf) of 50 kHz (determined on a liquid), a pulse length of 0.5 μs, 256 transients, and a recycle delay (rd) of 0.5 s. The ^31^P MAS-NMR experiments were obtained at 163 MHz with a rf of 100 kHz, a pulse length of 1.6 μs, 16 transients, and a rd of 120 s. The rd value was optimised on each sample to ensure quantitative measurements. The relative proportions between the different resonances of the ^31^P MAS-NMR spectra were obtained with the dmfit software [17]. The chemical shifts were referred to 0 ppm using Al(NO_3_)_3_ and H_3_PO_4_ solutions. The 1D correlation dipolar ^27^Al(^31^P) MAS-NMR Heteronuclear Multiple Quantum Coherence (D-HMQC [18,19,20,21]) experiment was used to produce a ^27^Al NMR spectrum showing only aluminium species interacting with P atoms through very short P/Al distance deriving from P–O–Al linkages. The experiment was performed with π/2-pulse of 9 and 5 μs on the ^27^Al and ^31^P channels, a 500 μs SR421 recoupling scheme, and 10 k transients separated by a rd of 0.5 s.

## 3. Results

**Figure 1:**
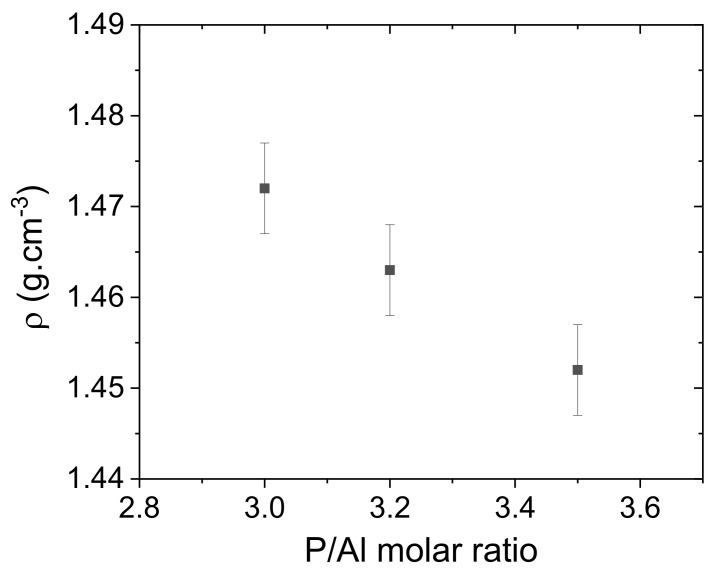
Density versus the P/Al molar ratio of the three solutions.

Data about the composition, P/Al molar ratio, P concentration [P], and density (ρ) are reported for the three solutions in Table 3. All the solutions are stable at room temperature (no precipitation was observed after several weeks of storage). As shown in Figure 1, the density values decrease when the P/Al ratio increases in agreement with the lower amount of introduced Al(OH)_3_ compound. The density values found in literature cannot be compared because the investigated solutions present lower P/Al ratio and/or higher [P] leading to much higher density values [3,13].

The XRD results are gathered in Table 4. Three compounds were identified in the XRD experiments (not shown here): cubic Al(PO_3_)_3_ [A], monoclinic Al(PO_3_)_3_ [B] [9], and AlPO_4_ (berlinite). No other phase was unambiguously identified.

**Figure 2:**
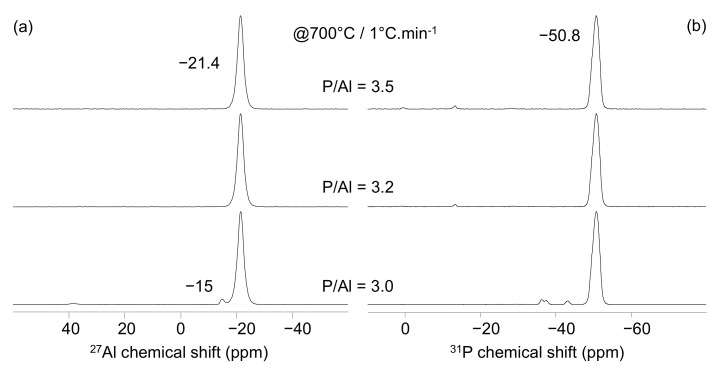
^27^Al (**a**) and ^31^P (**b**) MAS-NMR spectra obtained at 700 °C on Al/P = 3 (bottom), 3.2 (middle), and 3.5 (top) solutions.

The ^31^P and ^27^Al NMR spectra obtained on the three solutions treated at 700 °C (with a 1 °C/min heating rate) are reported in Figure 2. The ^27^Al NMR spectra present two signals in the chemical shift range corresponding to six-coordinated aluminate species (around −20 ppm) (Figure 2a). The three NMR experiments contain a high intensity peak centered at −21.4 ppm accompanied in the P/Al = 3 solution spectrum by a very low intensity peak at −15 ppm. The ^31^P NMR spectra show a high intensity peak at −50.8 ppm on the three spectra (Figure 2b). Low intensity peaks are also observed in the ^31^P NMR spectra at −36.5, −37.6, and −43.3 ppm in the P/Al = 3 solution and at 0 and −13 ppm in case of the P/Al = 3.2 and 3.5 solutions. The comparison between the chemical shift values observed in our study (given with an error of +/−0.1 ppm) and the data reported in Table 2 indicates that the binders obtained at 700 °C are mostly composed of cubic aluminium metaphosphate (Al(PO_3_)_3_ [A]). This phase is accompanied by low amounts of monoclinic aluminium metaphosphate when the binder is prepared with the P/Al = 3 molar ratio solution. These results are in a good agreement with the XRD experiments (Table 4). The presence of residual phosphoric acids is also highlighted when the binders are prepared from a solution with P/Al ratio higher than 3 on the ^31^P MAS-NMR spectra (narrow peaks around 0 and −13 ppm).

**Figure 3:**
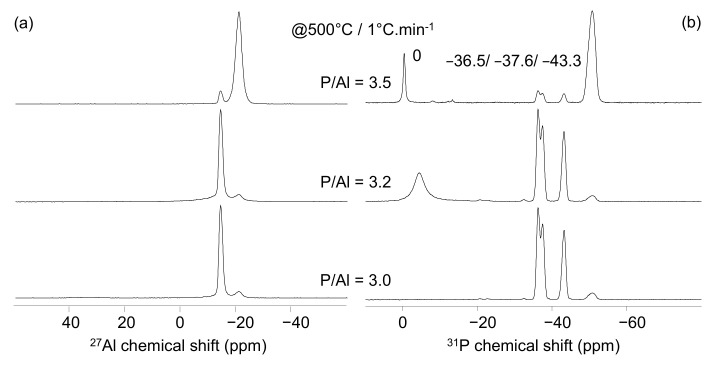
^27^Al (**a**) and ^31^P (**b**) MAS-NMR spectra obtained at 500 °C on Al/P = 3 (bottom), 3.2 (middle), and 3.5 (top) solutions.

The NMR analysis obtained on the binders prepared at 500 °C (with a 1 °C/min heating rate) are reported in Figure 3. The ^27^Al NMR spectra present the two peaks already observed in Figure 2a. However, while the −21.4 ppm peak is still the most important one for the P/Al = 3.5 solution, it becomes a low-intensity signal in the P/Al = 3.2 and 3 spectra that are dominated by the −15 ppm signal. The ^31^P NMR spectra also present identical peaks to those in Figure 2b. The −50.8 ppm signal is the highest intensity one in the P/Al = 3.5 spectra but not in the 3.2 and 3 spectra, for which the three peaks at −36.5, −37.6, and −43.3 ppm dominate. It is also noteworthy that a narrow peak at 0 ppm and a broad peak at −4.5 ppm are observed in the P/Al = 3.5 and 3.2 spectra, respectively. Based on the assignments reported in Table 2, our data show that the nature of the binders prepared at 500 °C is strongly related to the P/Al ratio. While the P/Al = 3.5 solution leads to a binder mostly composed of Al(PO_3_)_3_ [A], the two other binders contain Al(PO_3_)_3_ [B] as a major phase. As expected, residual phosphoric acid is found in the P/Al = 3.5 as free H_3_PO_4_. The broad peak (at −4.5 ppm) observed in case of the P/Al = 3.2 solution indicates the presence of phosphoric acid probably involved in hydrogen bonding.

**Figure 4:**
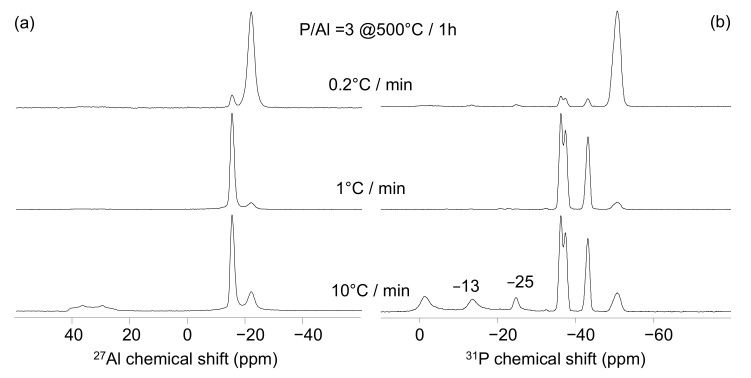
^27^Al (**a**) and ^31^P (**b**) MAS-NMR spectra obtained at 500 °C on the P/Al = 3 solution with 10 °C (bottom), 1 °C (middle), and 0.2 °C (top)/min heating rate. The dwell time is similar for the three experiments (1 h), leading to different total treatment times.

The ^27^Al and ^31^P NMR spectra obtained at 500 °C on the P/Al = 3 solution at different heating rates but with a constant dwell time of 1 h are presented in Figure 4. The two peaks at −21.4 and −15 ppm are present in the three ^27^Al NMR spectra (Figure 4a).

**Figure 5:**
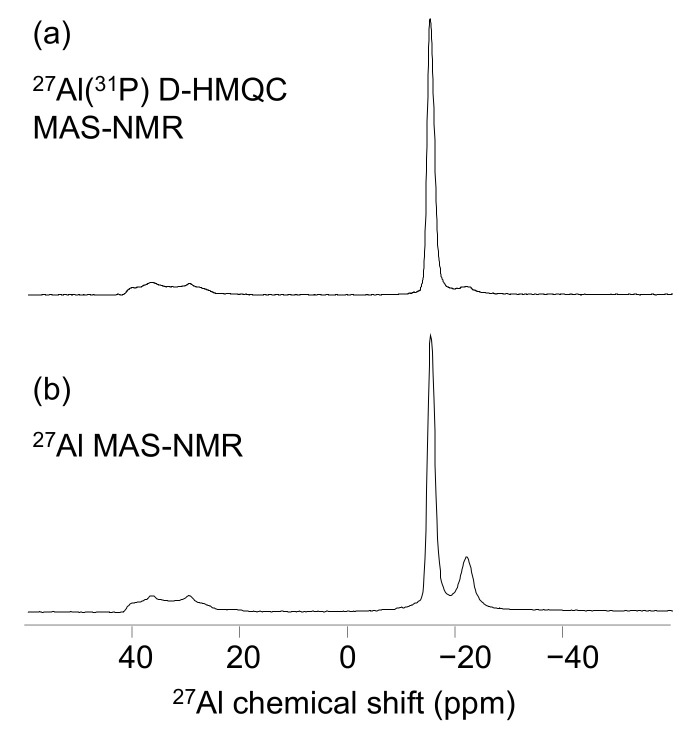
1D 27Al(31P) D-HMQC (**a**) and 27Al (**b**) MAS-NMR spectra obtained at 500 °C on the P/Al = 3 solution with 10 °C/min.

The −21.4 ppm signal dominates the 0.2 °C/min spectrum and the −15 ppm signal dominates the 1 and 10 °C/min spectra. This latter also contains an asymmetric signal, not observed in the previous samples, centered at 40 ppm, characteristic of four-coordinated aluminate species. The nature of that latter signal was investigated with the correlation 1D ^27^Al(^31^P) D-HMQC experiment. The obtained spectra is compared to the 1D classic spectrum (Figure 5a,b). The signal at 40 ppm (and the two others signals as well) is present in both the 1D and the 1D D-HMQC spectra, indicating that the three aluminate species belong to aluminophosphate compounds and do not originate to unreacted Al(OH)_3_. The comparison with the literature data [22] allows the assignment of this peak to berlinite AlPO_4_ in line with the XRD experiments (Table 4).

**Figure 6:**
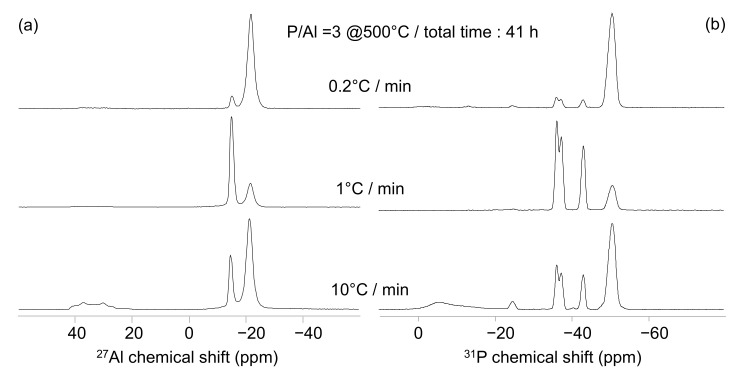
^27^Al (**a**) and ^31^P (**b**) MAS-NMR spectra obtained at 500 °C on the P/Al = 3 solution with 10 °C/(bottom), 1 °C (middle), and 0.2 °C (top)/min heating rate. The dwell times are different and lead to a similar total treatment time of 41 h for the three samples.

The ^31^P NMR spectra present the four peaks previously observed in Figure 2b and Figure 3b. The signal at −50.8 ppm dominates the spectrum recorded on the binder prepared at 0.2 °C/min, whereas the peaks at −36.5, −37.6, and −43.3 ppm dominate the spectra acquired on the binders prepared at 1 and 10 °C/min. The latter spectrum also contains broad peaks around −1 and −13 ppm as well as a narrow peak centered at −25.0 ppm in line with the presence of berlinite AlPO_4_ compound (Table 2) [22]. Our results show that the heating rate strongly modifies the nature of the binders obtained at 500 °C. The presence of Al(PO_3_)_3_ [A] is promoted by a low heating rate (0.2 °C/min), whereas 1 or 10 °C/min heating rates do not significantly change the Al(PO_3_)_3_ [B]/[A] ratio. However, a clear difference can be observed between these two heating rates with the presence of aluminium orthophosphate and residual phosphoric acids in case of the 10 °C/min protocol. The ^27^Al and ^31^P NMR spectra obtained at 500 °C on the P/Al = 3 solution with different heating rates but with a constant total treatment time of 41 h are presented in Figure 6. The ^27^Al MAS-NMR spectra present the three already mentioned peaks showing the presence of Al(PO_3_)_3_ [A], Al(PO_3_)_3_, [B] and AlPO_4_ (Figure 6a). Here again, the slow heating (0.2°/min) produces the binder with the maximum amount of stable Al(PO_3_)_3_ [A], indicating that heating rate is a key parameter of the thermal treatment. This conclusion is supported by the ^31^P MAS-NMR spectra where the signal corresponding to Al(PO_3_)_3_ [A] presents the maximum intensity for the slow heating (Figure 6b).

## 4. Discussion

### 4.1. Effect of the P/Al Molar Ratio

**Figure 7:**
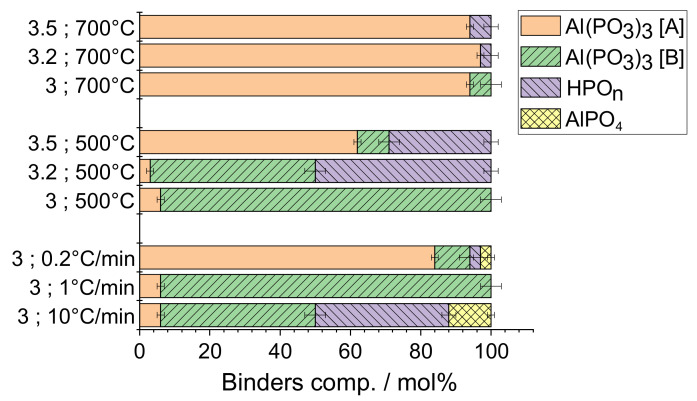
Binders compositions in mol% derived from the ^31^P MAS-NMR spectra decompositions. Only compounds with relative proportion higher than 1% were taken into account, and only binders prepared with a dwell time of 1 h are reported. Errors on the mol% are estimated to +/−3%.

In many applications, the formation of inorganic phosphate binders is achieved through the thermal treatment of the P/Al = 3 solution. This P/Al = 3 formulation is widely used [1,2,3,4,7,8,9,10,11,12,13,14] because it presents an identical P/Al ratio to that of the final binder component (cubic aluminium metaphosphate, Al(PO_3_)_3_ [A]). This ensures that no ‘free’ H_3_PO_4_ will be released during the thermal evolution. Indeed, H_3_PO_4_ release can be considered detrimental especially for the industrial devices used to treat the solution, and it can also interfere with other chemical compounds in the case of complex formulations. However, the solution with P/Al = 3.5 has shown a strong advantage related to the much lower curing temperature required to form the stable and final Al(PO_3_)_3_ [A] phase [10]. The first objective of this study was thus to determine if an intermediate formulation (P/Al = 3.2) will also present the same advantage of lower curing temperature combined to a lower amount of ‘free’ H_3_PO_4_. The molar composition of the binders obtained at 700 and 500 °C from the three solutions have been derived from the ^31^P MAS-NMR spectra decomposition (Table 5) and are reported in Figure 7 (only compounds with relative proportion higher than 1% were taken into account).

At 700 °C (Figure 7 top), there is not much difference between the three binders. All the three binders are mostly composed of the Al(PO_3_)_3_ [A] compounds. As previously reported [10], P in excess in the P/Al = 3.2 and 3.5 solutions allows for a complete transformation of the monoclinic to the cubic aluminium metaphosphate. The P/Al = 3.2 solution, presenting a lower amount of residual phosphoric acids, thus appears as the best formulation to prepare binders at high temperature. At 500 °C (Figure 7, middle), the nature of the binders is clearly different depending on the P/Al molar ratio of the heat-treated solution. The widely used P/Al = 3 solution produces a binder mostly composed of monoclinic Al(PO_3_)_3_ [B] (94%). As previously reported [10], the P/Al = 3.5 solution allows for the appearance of Al(PO_3_)_3_ [A] at lower temperature, leading to a binder containing 62% of cubic Al(PO_3_)_3_ but also large amounts (29%) of residual phosphoric acids. Surprisingly, the intermediate composition (P/Al molar ratio = 3.2) combines the presence of the monoclinic aluminium metaphosphate as a major phase (and thus the absence of cubic Al(PO_3_)_3_) and the presence of residual phosphoric acids in high proportion (50%). The corresponding broad peak on the ^31^P MAS-NMR spectrum suggests that the residual HPO_n_ is linked through hydrogen bonding, which could explain why it does not evolve from the material at 500 °C (Figure 3b). Our study shows that the P/Al = 3.2 formulation should be preferred in applications involving high-temperature treatments. However, this solution is not suitable for low temperature applications in which better results are obtained with the P/Al = 3.5 solution.

### 4.2. Effect of The Heating Rate

In numerous papers devoted to the alumino-phosphate solution chemistry, the thermal treatment is only defined with the final curing temperature with no real information about the heating rate [1,4,11,12,14]. However, our results obtained on the P/Al = 3 solution treated 1 h at 500 °C with different heating rates clearly show that this parameter has a strong impact on the binder nature (Figure 7, bottom). As previously indicated, the binder obtained with a heating rate of 1 °C/min was composed of 6% of cubic and 94% of monoclinic aluminium metaphosphate. A much lower heating rate (0.2°/min) allowed for a better evolution from the monoclinic to the cubic Al(PO_3_)_3_ compound. The binder appears thus to be composed of more than 85% of Al(PO_3_)_3_ [A] compounds. Surprisingly, the two Al(PO_3_)_3_ compounds are accompanied by residual HPO_n_ and AlPO_4_ in low proportions (3%). This indicates that the mixed alumino-phosphate species react to create free phosphate moieties (leading to HPO_n_) and lower P/Al ratio units (leading to AlPO_4_ compound). This behaviour also occurs in case of a high heating rate (10°/min) but on a much more important scale, with almost 40% of the binder being composed of HPO_n_. Moreover, the cubic aluminium metaphosphate is not dominant compared to the monoclinic compound. Fast heating does not promote the formation of stable binder and should thus be replaced by slow heating protocol. Of course, the heating rate has a direct impact on the global treatment time, with slow heating producing longer treatment times. However, the results reported in Figure 6, where the total treatment times are similar (41 h), clearly show that the heating rate is a key parameter. The binders prepared with the slow heating contain the maximum amount of stable cubic aluminium metaphosphate. Therefore, we do believe that slow heating should be preferred to ensure the formation of stable binders at low temperatures.

## 5. Conclusions

A new alumino-phosphate solution with a molar P/Al ratio of 3.2 was prepared, and its efficiency in preparing stable binders at 500 and 700 °C (using a heating rate of 1 °C/min) was tested. Our results confirm that P in excess (P/Al > 3) decreases the appearance temperature of the stable cubic aluminium metaphosphate Al(PO_3_)_3_[A]. Compared to the P/Al = 3.5 solution, the P/Al = 3.2 solution presents an advantage in the preparation of the binder at 700 °C, because of the lower amount of ‘free’ HPO_n_. However, no advantage was observed for preparing binders at 500 °C. The effect of heating rates was tested on the widely used P/Al = 3 solution to prepare binders at 500 °C. It turns out that the proportion of Al(PO_3_)_3_ [A] is higher when the solution is treated with slow heating. We believe that this parameter is critical in the description of the thermal treatment of the solution and should be systematically mentioned in the respective protocols.

## Figures and Tables

**Table 1:** The chemical structure and temperature domains of compounds involved in the thermal evolution of P/Al = 3 solution (from [10]).

T/°C	Compounds
150–250 °C	Al(H_2_PO_4_)_3_
350 °C	AlH_2_P_3_O_10_, × H_2_O
500 °C	Al(PO_3_)_3_ [B] + [A]
1000 °C	Al(PO_3_)_3_ [A]

**Table 2:** ^31^P and ^27^Al NMR chemical shift values of the Al_2_O_3_-P_2_O_5_-H_2_O compounds observed during the thermal evolution of the P/Al = 3 solution (from [10,15,16]).

	δ(^31^P) (ppm)	δ(^27^Al) (ppm)
**Al(PO_3_)_3_ [A]**	−50.8	−21.4
**Al(PO_3_)_3_ [B]**	−36.5/−37.6/−43.3	−15
**AlH_2_P_3_O_10_, × H_2_O**	−20.9/−22.9/−32.5	−13.2/−15.2
**Al(H_2_PO_4_)_3_**	−15.8	−16.5
**Phosphoric Acids HPO_n_**	0, −13	-

**Table 3:** Solution preparation parameters (mass of Al(OH)_3_, volumes of H_3_PO_4_ and H_2_O, concentration of P ([P])) and density (ρ). The error on the density is +/− 0.005.

P/Al	mAl(OH)_3_ (g)	vol H_3_PO_4_/H_2_O (mL/ mL)	[P] (g.mol^−1^)	ρ (g.cm^−3^)
3	18.936	50/50	7.41	1.472
3.2	17.753	50/50	7.41	1.463
3.5	16.458	50/50	7.41	1.452

**Table 4:** Phases identified on the XRD experiments. Final T: final temperature. Only the data obtained on binders prepared with a dwell time of 1 h are reported here.

P/Al; Final T; Heating Rate	Major Compound	Minor Compound
3; 700 °C; 1 °C/min	Al(PO_3_)_3_ [A]	Al(PO_3_)_3_ [B]
3.2; 700 °C; 1 °C/min	Al(PO_3_)_3_ [A]	-
3.5; 700 °C; 1 °C/min	Al(PO_3_)_3_ [A]	-
3; 500 °C; 1 °C/min	Al(PO_3_)_3_ [B]	Al(PO_3_)_3_ [A]
3.2; 500 °C; 1 °C/min	Al(PO_3_)_3_ [B]	Al(PO_3_)_3_ [A]
3.5; 500 °C; 1 °C/min	Al(PO_3_)_3_ [A]	Al(PO_3_)_3_ [B]
3; 500 °C; 0.2 °C/min	Al(PO_3_)_3_ [A]	Al(PO_3_)_3_ [B]
3; 500 °C; 10 °C/min	Al(PO_3_)_3_ [B]	Al(PO_3_)_3_ [A], AlPO_4_ (berlinite)

**Table 5:** Percentage of P signals deduced from the ^31^P NMR spectra decompositions. Final T: final temperature. The errors on the quantification are +/−1%. Only the data obtained on binders prepared with a dwell time of 1 h are reported here.

	% of total P (+/−1) involved in			
P/Al; Final T; Heating Rate	Al(PO_3_)_3_ [A]	Al(PO_3_)_3_ [B]	HPO_n_	AlPO_4_
3; 700 °C; 1 °C/min	94	6	-	-
3.2; 700 °C; 1 °C/min	99	-	1	-
3.5; 700 °C; 1 °C/min	98	-	2	-
3; 500 °C; 1 °C/min	6	94	-	-
3.2; 500 °C; 1 °C/min	4	71	25	
3.5; 500 °C; 1 °C/min	77	11	12	-
3; 500 °C; 0.2 °C/min	84	10	3	3
3; 500 °C; 10 °C/min	9	66	19	6

## Data Availability

Data sharing is not applicable for this paper.
